# Role of ferroptosis and ferroptosis-related non-coding RNAs in the occurrence and development of gastric cancer

**DOI:** 10.3389/fphar.2022.902302

**Published:** 2022-08-15

**Authors:** Ling Lu, Bei Chen, Yumeng Xu, Xinyi Zhang, Longtao Jin, Hui Qian, Yi Wang, Zhao Feng Liang

**Affiliations:** ^1^ Child Healthcare Department, The Fourth Affiliated Hospital of Jiangsu University, Zhenjiang, JS, China; ^2^ Jiangsu Key Laboratory of Medical Science and Laboratory Medicine, School of Medicine, Jiangsu University, Zhenjiang, JS, China; ^3^ Suzhou Science and Technology Town Hospital, Suzhou, JS, China; ^4^ Department of Urology, the Second Hospital of Anhui Medical University, Hefei, China

**Keywords:** gastric cancer, ferroptosis, noncoding RNA, mechanisms, function

## Abstract

Gastric cancer (GC) is a malignant cancer of the digestive tract and is a life-threatening disease worldwide. Ferroptosis is a newly discovered form of regulated cell death, which involves the accumulation of iron-dependent lipid peroxides. It has been found that ferroptosis plays an important regulatory role in the occurrence, development, drug resistance, and prognosis of GC. Non-coding RNAs (ncRNAs) play a critical role in the occurrence and progression of a variety of diseases including GC. In recent years, the role of ferroptosis and ferroptosis-related ncRNAs (miRNA, lncRNA, and circRNA) in the occurrence, development, drug resistance, and prognosis of GC has attracted more and more attention. Herein, we briefly summarize the roles and functions of ferroptosis and ferroptosis-related ncRNAs in GC tumorigenesis, development, and prognosis. We also prospected the future research direction and challenges of ferroptosis and ferroptosis-related ncRNAs in GC.

## Introduction

Cell death is strictly regulated by complex intracellular and extracellular signals, and is very important for various physiological and pathological processes, including growth, development, and tumorigenesis. The imbalance between abnormal proliferation and cell death of cancer cells is an important basis for the biological characteristics of malignancy. Ferroptosis is a newly discovered form of regulated cell death, which involves the accumulation of iron-dependent lipid peroxides and leads to fatal cell damage (Zhang et al., 2020). Ferroptosis, a unique form of nonapoptotic-regulated cell death caused by overwhelming iron-dependent lipid peroxides, is considered an emerging cancer suppression mechanism for gastric cancer (GC) (Ma et al., 2022).

GC is one of the most common malignant cancers that seriously affect human health in the world. Although great progress has been made in the diagnosis and treatment of GC in recent years, there is still lack of effective diagnostic markers, and the prognosis of advanced GC is not optimistic. Studies have started to reveal the essential role of ferroptosis in GC (Lee et al., 2020; Zhang et al., 2020; Guan et al., 2022; Ma et al., 2022). Ferroptosis plays an important regulatory role in the occurrence, development, invasion, migration, diagnosis, drug resistance, and prognosis of GC (Gomaa et al., 2019; Zhang et al., 2020; Liu Y. et al., 2021; Guan et al., 2022). Increasing evidence has shown that non-coding RNA (ncRNAs) play a crucial role in the occurrence and development of GC. NcRNAs are important regulators of gene expression and contribute to the promotion of a large number of human diseases. In general, miRNAs negatively regulate gene expression by binding to the 3′ untranslated region of the target messenger RNAs (mRNAs), resulting in mRNA silencing or degradation (Luo et al., 2019; Yan and Bu, 2021). Long noncoding RNAs (lncRNAs) are a class of potentially-coding RNA transcripts, which have the functions of regulating gene expression by inhibiting the mRNA translation, modulating the mRNA stability, or as competing endogenous RNAs (ceRNAs) by acting as miRNA sponges (Li et al., 2020a; Yan and Bu, 2021). Circular RNAs (circRNAs) are mainly produced by the reverse splicing of exons of precursor mRNAs. The functions of circRNAs are mainly ceRNA or miRNA sponging, binding with proteins, regulation of precursor mRNAs (pre-mRNA) splicing, regulation of parental gene expression, and potential translation templates for proteins or peptides (Yan and Bu, 2021; Zhang Y. et al., 2022). MiRNAs, lncRNAs, and circRNAs may have other ways to regulate the expression of mRNAs or proteins jointly or competitively. NcRNAs play a key role in GC occurrence and development with disruption of their function including gene splicing and transcription as well as biological processes related to ferroptosis, cell differentiation, migration, apoptosis, and immune response (Wei et al., 2020; Tang et al., 2021; Ye et al., 2021).

However, the role of ncRNAs associated with ferroptosis in GC has not been systematically discussed. Herein, we analyzed and discussed the relationship between ferroptosis, ferroptosis-related ncRNAs, and GC. This review summarized the role of ferroptosis and ferroptosis-related ncRNAs in the occurrence, development, drug resistance, and prognosis of GC, which may provide a new basis for the early diagnosis and treatment of GC.

## Ferroptosis and gastric cancer

Ferroptosis is a newly defined form of programmed cell death characterized by iron-dependent peroxide lipid accumulation (Shao et al., 2021). Fe^3+^ enters cells and is reduced to Fe^2+^ through STEAP3. Then, the divalent metal transporter 1 (DMT1) leads to the release of Fe^2+^ from endosomes, leading to the accumulation of ROS, which induce lipid peroxidation and ferroptosis (Jiang N. et al., 2021; Zhang C. et al., 2022). Glutathione peroxidase 4 (GPX4)-mediated lipid peroxidation pathway plays an important role in inhibiting ferroptosis. GPX4 converts glutathione (GSH) into oxidized glutathione (GSSG) and reduces lipid peroxidation (Jiang N. et al., 2021; Zhang C. et al., 2022).

GC is a malignant tumor that causes a great burden globally, and its molecular mechanism is not very clear. Dysregulation of the balance between cell proliferation and death is a central feature of GC. Studies have found that ferroptosis plays a critical role in the carcinogenesis and progression of GC (Gomaa et al., 2019; Liu Y. et al., 2021). The levels of ferroptosis were related to a variety of prognosis and cancer immune characteristics, which might be conducive to the individualized treatment of GC (Ma et al., 2021).

Ferroptosis played a regulatory role in GC by affecting the biological characteristics of GC cells, such as proliferation, migration, and apoptosis. Ferritinophagy-induced ferroptosis and the KEAP1/NRF2*/*HO-1 pathway was involved in the epithelial-to-mesenchymal transition (EMT) process of GC cells (Guan et al., 2022). Lee et al. demonstrated that the biosynthetic pathway of polyunsaturated fatty acids determines the sensitivity of GC to ferroptosis (Lee et al., 2020). The data of Sun et al. expounded that perilipin-2 promotes the proliferation or apoptosis of GC cells by modifying the ferroptosis pathway (Sun et al., 2020). It was reported that cytoplasmic polyadenylation element binding protein 1 (CPEB1) enhanced erastin-induced ferroptosis in GC cells by inhibiting TWIST1, and then promotes GC cells metastasis and angiogenesis (Wang J. et al., 2021). The above studies showed that ferroptosis plays an important role in the occurrence and development of GC, and its specific role and mechanism need to be further explored.

The drug resistance of patients with advanced GC seriously affects the effect of chemotherapy. Many studies suggested that ferroptosis could enhance the sensitivity of GC cells to chemotherapeutic drugs. Zhang et al. demonstrated that miR-522 secreted by cancer-associated fibroblasts (CAFs) suppressed ferroptosis and promoted chemoresistance in GC (Zhang et al., 2020). The results revealed that apatinib could induce lipid peroxidation through SREBP-1a-mediated GPX4, and regulate multidrug resistance and ferroptosis of GC cells (Zhao et al., 2021). Activating transcription factor 3 (ATF3) made GC cells sensitive to cisplatin by blocking NRF2*/*KEAP1*/*XCT pathway transduction and inducing ferroptosis, which provides a promising treatment for overcoming the chemoresistance of GC (Fu et al., 2021). The silent information regulator 6 (SIRT6) silencing overcomes resistance to sorafenib via promoting ferroptosis in GC (Cai et al., 2021).

Ferroptosis was expected to be used in the treatment and prognosis of GC (Liu S. J. et al., 2021; Huo et al., 2021). Shao et al. screened 10 ferroptosis-related markers (SP1, MYB, ALDH3A2, KEAP1, AIFM2, ITGB4, TGFBR1, MAP1LC3B, NOX4, and ZFP36), which could well predict the prognosis and immunotherapy of GC (Shao et al., 2021). Studies have shown that by changing the activation degree of ferroptosis, GC cells and the microenvironment can be formed (Xiao et al., 2021a). Ferroptosis-related genes *NOX4, CHAC1,* and *HIF1A* were the prognostic biomarkers of gastric adenocarcinoma (Xiao et al., 2022). In addition, it was also found that the ferroptosis pattern in GC is related to the characteristics of immune microenvironment (Jiang X. et al., 2021; Liu S. J. et al., 2021; Wang F. et al., 2021). The establishment of markers associated with ferroptosis will help to predict the biological characteristics of GC and select the appropriate treatment for GC patients. However, there are still many problems to be solved in the application of ferroptosis to the clinical diagnosis and treatment of GC.

Ferroptosis was the major mechanism mediating antitumor activity, which was expected to become a promising compound for the treatment of GC (Liu Y. et al., 2021; Zhang L. et al., 2022; Ye et al., 2022). Jiyuan oridonin A, a naturally occurring ent-kaurane diterpenoid, induced ferroptosis through the autophagy pathway, suggesting that the induction of ferroptosis may be the main mechanism mediating the antitumor activity of Jiyuan oridonin A and its derivatives (Liu Y. et al., 2021). The results showed that Yiqi Huayu Decoction (a Chinese herbal medicine formula) could induce GC ferroptosis through the JAK2-STAT3 pathway and ACSl4 (Song et al., 2022). The data of Zhang et al. suggested that 6-Thioguanine (6-TG) performed as a potential novel compound for GC treatment via inducing ferroptosis (Zhang J et al., 2022). Tanshinone IIA, a pharmacologically active component isolated from Chinese herb, induced ferroptosis in GC cells by affecting the expression of p53-mediated SLC7A11 (Guan et al., 2020). Ma et al. demonstrated that the activating MAT2A-ACSL3 pathway could protect GC cells from ferroptosis2. However, it is not clear how ferroptosis plays a crucial role in the occurrence, development, and diagnosis of GC. Recent studies have found that ncRNAs may play a key role in this process.

## Ferroptosis-related non-coding RNA and gastric cancer

More and more evidences have shown that ferroptosis-related ncRNAs play critical roles in the occurrence, development, treatment, and prognosis of GC. We summarized and analyzed the role of ferroptosis-related ncRNAs in the occurrence, progression, and drug resistance of GC.

### Ferroptosis-related miRNA and gastric cancer

Ferroptosis is an iron-dependent mediated necrosis, which plays a decisive role in the occurrence and development of GC. It is reported that miRNAs play an important role in the multiple stages of GC (Li et al., 2020b; Wang J. et al., 2022). Studies suggested that miRNAs regulate the ferroptosis process of GC cells. We summarized the role of ferroptosis-related miRNAs in the occurrence, development, prognosis, and drug resistance of GC*.*

ALOX15 was closely related to the production of lipid peroxidation in GC cells, and miR-522 was a potential inhibitor of ALOX15. Zhang et al. demonstrated that CAFs secrete exosomal miR-522 to inhibit ferroptosis by blocking ALOX15 and lipid peroxidation accumulation (Zhang et al., 2020). GC stem cells are the main cause of metastasis and drug resistance of GC. It was found that the miR-375/SLC7A11 axis could stimulate ferroptosis, thus reducing the stemness of GC cells (Ni et al., 2021). The study of Niu et al. confirmed that physcion 8-O-β-glucopyranoside plays an important role in promoting ferroptosis by regulating miR-103a-3p/GLS2, so as to highlight its therapeutic potential in GC (Niu et al., 2019). Levobupivacaine has potential anticancer properties. Levobupivacaine, a local anesthetic, induced ferroptosis of GC cells and inhibited the growth of GC cells through the miR-489-3p/SLC7A11 axis (Mao et al., 2021). Propofol can inhibit the proliferation and induce the apoptosis of GC cells. Propofol induced ferroptosis and inhibited malignant phenotypes of GC cells via the miR-125b-5p/STAT3 axis (Liu Y. P. et al., 2021). The data of Gomaa et al. identified a new mechanism mediating miR-4715-3p silencing and AURKA induction in upper gastrointestinal adenocarcinoma. The inhibition of AURKA or reconstitution of miR-4715-3p inhibited GPX4 and induced cell death, suggesting a link between AURKA and ferroptosis (Gomaa et al., 2019).

Ferroptosis has been proved to play an important role in the pathogenesis of GC. MiRNAs have regulatory function in GC cells and have potential diagnostic and prognostic value in the occurrence and development of GC. Ferroptosis-related miRNAs have potential clinical application prospect in the diagnosis, personalized treatment, and prognosis of GC (Table 1).

**TABLE 1:** Overview of the role of ferroptosis-related miRNAs in GC.

MiRNA	Expression status	Relationship with autophagy	Target	Type of biomarker	References
miR-522	Upregulated	Inhibited ferroptosis	ALOX15	Chemo- resistance	Zhang et al. (2020)
miR-375	Upregulated	Triggered ferroptosis	SLC7A11	Stemness of GC	Ni et al. (2021)
miR-103a-3p	Downregulated	Promoted ferroptosis	GLS2	Development and prognosis	Niu et al. (2019)
miR-489-3p	Upregulated	Enhanced ferroptosis	SLC7A11	Development and treatment	Mao et al. (2021)
miR-125b-5p	Upregulated	Enhanced ferroptosis	STAT3	Development	Liu Y. P. et al. (2021)
miR-4715-3p	Downregulated	Enhanced ferroptosis	AURKA/GPX4	Development and prognosis	Gomaa et al. (2019)

### Ferroptosis-related lncRNA and gastric cancer

LncRNAs and ferroptosis play a crucial role in the occurrence and development of GC (Jiang N. et al., 2021). In this part, we focus on the role and mechanism of ferroptosis-related lncRNAs in the occurrence and development of GC.

It is reported that the lncBDNF-AS/WDR5/FBXW7 axis regulated ferroptosis and mediated peritoneal metastasis of GC through VDAC3 ubiquitination (Huang et al., 2022). The experiment of Wang et al. confirmed that lncLASTR mediated the proliferation and migration of GC cells through the regulation of ferroptosis (Wang G. et al., 2022). GC stem cells (GCSC) are the main cause of the occurrence and prognosis of GC. Zhang et al. suggested that GC cell-derived exosomal lncFERO controls the tumorigenicity of GCSC by inhibiting ferroptosis, suggesting that the targeted exosomal lncFERO/hnRNPA1/SCD1 combined chemotherapy may be a promising GCSC based therapeutic strategy (Zhang et al., 2021). Yao et al. found that lncRNAs (A2M-AS1, C2orf27A, and ZNF667-AS1) targeted ferroptosis-related genes and impaired the activation of CD4^+^ T cells in GC, which provides a new strategy of GC immunotherapy (Yao et al., 2021). *Pan* et al. prognostic model based on the 17 ferroptosis-related lncRNAs may improve the overall survival prediction of GC (Pan et al., 2021). These ferroptosis-related lncRNAs may play an important role in the immune infiltration of GC, which may help to determine the personalized prognosis and treatment of GC patients (Chen W. et al., 2021). Zhang et al. also established a ferroptosis-related lncRNA model that could predict the prognosis of stomach adenocarcinoma patients (Zhang S. et al., 2022). There are other prediction models showing ferroptosis-related lncRNAs associated with drug resistance, immunity, and tumor microenvironment changes in GC (Chen X. et al., 2021; Lai et al., 2021; Xiao et al., 2021b; Wei et al., 2021; Zhang S. et al., 2022).

Ferroptosis-related lncRNAs are differentially expressed in different stages of GC, which can provide a basis for ferroptosis-related lncRNAs clinical application in the diagnosis, treatment, and prognosis of GC. These studies suggest that ferroptosis-related lncRNAs can be used as potential markers for the progression, prognosis, personalized treatment, and drug resistance of GC (Table 2).

**TABLE 2:** Overview of the role of ferroptosis-related lncRNAs in GC.

LncRNA	Expression status	Relationship with autophagy	Target	Type of biomarker	References
lncBDNF-AS	Upregulated	Inhibited ferroptosis	WDR5/FBXW7	Development	Huang et al. (2022)
lncLASTR	Upregulated	Inhibited ferroptosis	GPX4	Occurrence, development, and prognostic	Wang G. et al. (2022)
lncFERO	Upregulated	Inhibited ferroptosis	hnRNPA1/SCD1	Development, resistance, and treatment	Zhang et al. (2021)
lncA2M-AS1, C2orf27A, and ZNF667-AS1	Upregulated	Inhibited ferroptosis	hub FRGs (MYB, PSAT1, TP53, and LONP1)	Treatment and prognostic	Yao et al. (2021)
17 ferroptosis-related lncRNAs	Differential expression			Treatment and prognostic	Pan et al. (2021)
20 ferroptosis-related lncRNAs	Differential expression			Diagnostic and prognostic	Chen W. et al. (2021)
AP000695.2, AL365181.3, and LINC01615	Upregulated			Treatment and prognostic	Zhang S. et al. (2022)
AP003392.1, AC245041.2, AP001271.1, and BOLA3-AS1	Expression correlated with clinical features			Treatment and prognostic	Wei et al. (2021)
17 ferroptosis-related lncRNAs	Differential expression		cytoskeleton, WNT and PI3K/mTOR	Development, treatment, and prognostic	Xiao et al. (2021b)
10 ferroptosis-related lncRNAs	Differential expression			Prognostic and resistance	Lai et al. (2021)
TMEM105, PVT1, LOC646588, FLJ22447, and DLEU1	Differential expression			Treatment and prognostic	Chen X. et al. (2021)

### Ferroptosis-related circRNAs and gastric cancer

Multiple circRNAs have been verified to act as essential regulators in the occurrence and progression of GC. As new prognostic biomarkers, ferroptosis-related circRNAs will be broad application prospects in GC diagnosis, individualized treatment, microenvironment, drug resistance, and immunotherapy in the future. Herein, we summarized the role of ferroptosis-related circRNAs in GC.

The expression level of circ0008035 was upregulated in GC tissues and cells. Li et al. reported that circ0008035 repressed ferroptosis and affected the proliferation and apoptosis of GC cells by up-regulating *EIF4A1* via sponging miR-599 (Li C. et al., 2020). Circ0000190 was down regulated in GC tissues and cell lines, indicating a poor prognosis of GC patients. Circ0000190 overexpression suppressed GC cell proliferation and migration by inducing ferroptosis (Jiang et al., 2022).

## Summary and the challenges

**FIGURE 1:**
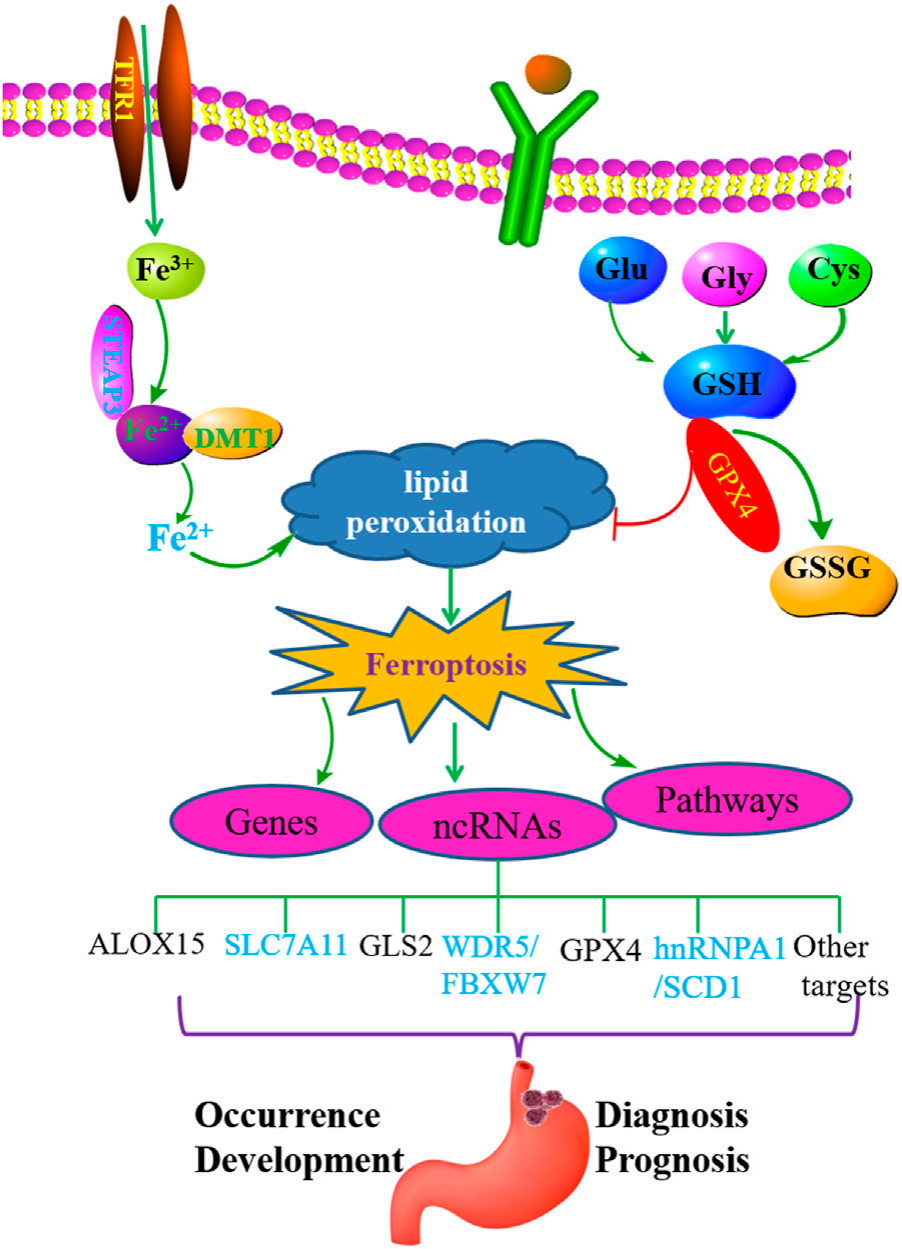
Ferroptosis plays an important role in GC and may regulate the occurrence and development of gastric cancer by interacting with related ncRNAs.

Ferroptosis plays an important role in the pathogenesis of GC. NcRNAs play a crucial role in the occurrence, development, treatment, and drug resistance of GC. However, the relationship between ferroptosis, ferroptosis-related ncRNAs, and GC has not been well summarized and clarified. In recent years, the understanding of the relationship between ferroptosis, ferroptosis-related ncRNAs, and GC has advanced rapidly. Based on the abovementioned overview , we conclude that ferroptosis and ferroptosis-related ncRNAs play essential roles in the occurrence and prognosis of GC (Figure 1). Ferroptosis-related ncRNAs are expected to be used as potential clinical markers of GC.

However, to achieve clinical application, there are still many aspects to be improved and many challenges to be solved in the future research. First, the regulatory mechanism between ferroptosis and ferroptosis-related ncRNAs are not very clear. In the future, we believe that the role and mechanism of ferroptosis-related ncRNAs in GC may become one of the research focuses. Second, the mechanisms of generation and selection of ferroptosis-related ncRNAs are still unclear. Clarifying the mechanism of ferroptosis ncRNA generation, selection and degradation may be an important link in promoting its clinical application. Third, at present, there are only few studies on ferroptosis-related ncRNAs in the occurrence and prognosis of GC. It is necessary to further explore the role of more ncRNAs in GC. Furthermore, the research and verification of large-scale population tissue samples need to be carried out before clinical application. The reproducibility, specificity, and sensitivity of ferroptosis-related ncRNA detection and application need to be further evaluated. In addition, exosomes can carry mRNAs, ncRNAs, proteins, and other components to participate in the cell-cell communication. Whether ferroptosis related ncRNAs can affect the microenvironment of GC through exosomes, and then play key roles in the occurrence, prognosis, and drug resistance of GC needs to be explored.

Based on the abovementioned studies, we also speculated the potential future development direction of ferroptosis and ferroptosis-related ncRNAs in GC. First, developing experimental methods or detection techniques of ferroptosis and ferroptosis-related ncRNAs, so that they can be better used in the early diagnosis of GC, monitoring progress, drug resistance, and prognosis. Second, liquid biopsy is more and more widely used in GC and other cancers. It is particularly important to detect ferroptosis-related ncRNAs in blood or exosomes, and analyze the relationship between these abnormally expressed ncRNAs and the occurrence, development, drug resistance, and prognosis of GC. Last, as a potential therapeutic target for GC, giving better use of ferroptosis-related ncRNAs in diagnosis and treatment may help to prolong the survival time and improve the quality of life of GC patients.
